# Beneficial Effects of Naringenin in Cigarette Smoke-Induced Damage to the Lung Based on Bioinformatic Prediction and In Vitro Analysis

**DOI:** 10.3390/molecules25204704

**Published:** 2020-10-14

**Authors:** Pan Chen, Ziting Xiao, Hao Wu, Yonggang Wang, Weiyang Fan, Weiwei Su, Peibo Li

**Affiliations:** Guangdong Engineering and Technology Research Center for Quality and Efficacy Re-evaluation of Post-marketed TCM, Guangdong Key Laboratory of Plant Resources, School of Life Sciences, Sun Yat-sen University, Guangzhou 510275, China; chenpan989@126.com (P.C.); xiaoziting@hotmail.com (Z.X.); wuhao_cpu@126.com (H.W.); wangyg@mail.sysu.edu.cn (Y.W.); fanwy5@mail2.sysu.edu.cn (W.F.); lsssww@mail.sysu.edu.cn (W.S.)

**Keywords:** naringenin, lung health, cigarette smoke, oxidative stress, inflammation, system bioinformatic approach, dynamic change, Nrf2 pathway

## Abstract

Naringenin is found mainly in citrus fruits, and is thought to be beneficial in the prevention and control of lung diseases. This study aims to investigate the mechanisms of naringenin against the damage in the lung caused by cigarette smoke. A system bioinformatic approach was proposed to predict the mechanisms of naringenin for protecting lung health. Then, we validated this prediction in BEAS-2B cells treated with cigarette smoke extract (CSE). System bioinformatic analysis indicated that naringenin exhibits protective effects on lung through the inhibition of inflammation and suppression of oxidative stress based on a multi-pathways network, mainly including oxidative stress pathway, Nrf2 pathway, Lung fibrosis pathway, IL-3 signaling pathway, and Aryl hydrocarbon receptor pathway. The in vitro results showed that naringenin significantly attenuated CSE-induced up-regulation of IL-8 and TNF-α. CSE stimulation increased the mRNA expressions of Nrf2, HO-1, and NQO1; the levels of total protein and nuclear protein of Nrf2; and the activity of SOD on days 2 and 4; but decreased these indexes on day 6. Naringenin can balance the antioxidant system by regulating Nrf2 and its downstream genes, preliminarily validating that Nrf2 pathway is involved in the protection offered by naringenin against cigarette smoke-induced damage to the lung. It suggests that dietary naringenin shows possible potential use in the management of lung health.

## 1. Introduction

Hundreds of millions of people suffer from lung diseases each year, resulting in four million premature deaths [1]. The report identifies five conditions including asthma, chronic obstructive pulmonary disease (COPD), acute respiratory infections, tuberculosis, and lung cancer, which are large contributors to the leading causes of death and the global burden of respiratory disease [2]. Not enough effective therapies have been made for solving these illnesses to date, despite most major respiratory illnesses being preventable [3]. Cigarette smoke (CS), air pollution, and occupational exposures to lung toxins are the leading factors responsible for the major lung diseases [4]. More than any other tissue, the lung is susceptible to oxidative changes in protein function expression and structure. One of the greatest challenges to lung cell proteostasis is exposure of the respiratory epithelium to cigarette smoke [5]. Cigarette smoke, including passive smoke exposure, is a leading cause of chronic COPD and other lung diseases [6], along with air pollution and workplace exposure to noxious air. COPD is the third leading cause of death globally, and its global burden continues to grow [7]. By far the most common cause of COPD is cigarette smoke, with several other factors such as air pollution and genetic factors causing or worsening COPD [8]. The overall incidence of COPD is significantly higher in smokers [9]. Additionally, cigarette smoking is the major cause of lung cancer [10]; cigarette smoke exposure during pregnancy is harmful to fetal development and increases the risk of childhood asthma [11]. Obviously, cigarette smoking has already caused serious lung health problems. On the one hand, cigarette smoke would induce the release of proinflammatory cytokines and promote the recruitment of lymphocytes, neutrophils, and eosinophils to the respiratory tract, eventually leading to airway inflammation [12,13]; on the other hand, it would disturb the oxidant/antioxidant balance, leading to cellular damage in the respiratory tract.

Naringenin, a natural bioactive flavonoid, is found mainly in citrus fruits [14]. Accumulating evidence has suggested that naringenin could inhibit inflammatory response and oxidative stress; Podder et al. [15] found that naringenin exerted cytoprotective effect against paraquat-induced toxicity in human bronchial epithelial BEAS-2B cells by activating Nrf2; Fan et al. [16] found that naringenin exerted anti-inflammatory effects by downregulating NF-κB and activating the Nrf-2/HO-1 pathway. There is increasing evidence that naringenin is thought to be beneficial in lung health. It has been reported that naringenin has a protective effect on airway inflammatory diseases including COPD, asthma, lung cancer, pulmonary fibrosis, and cystic fibrosis in different experimental models [17]. Liu et al. [18] investigated the effects of naringenin on COPD caused by CS in BALB/c mouse and A549 cells, finding that naringenin could inhibit the COPD-related inflammation. Therefore, dietary naringenin shows promise as a functional food for protecting against the damage in the lung caused by cigarette smoke, and the mechanisms of naringenin leading to these protective effects need to be studied systematically.

The present study aims to predict the potential mechanism of naringin in the prevention and control of lung diseases through an integrative system bioinformatic approach. Then, we investigated the CSE-induced dynamic change of inflammation and oxidative/antioxidant system in BEAS-2B cells to preliminarily validate the mechanisms.

## 2. Results

### 2.1. PPI Network and Function Module Analysis

**Figure 1:**
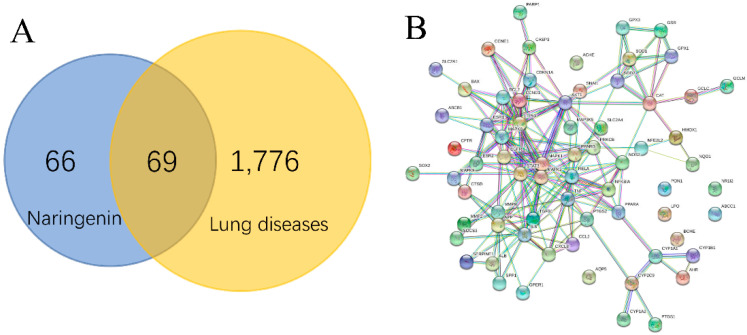
Interaction targets of naringenin and lung diseases in Venn diagram (**A**). PPI network of interaction targets for naringenin-treated lung diseases (**B**).

**Figure 2:**
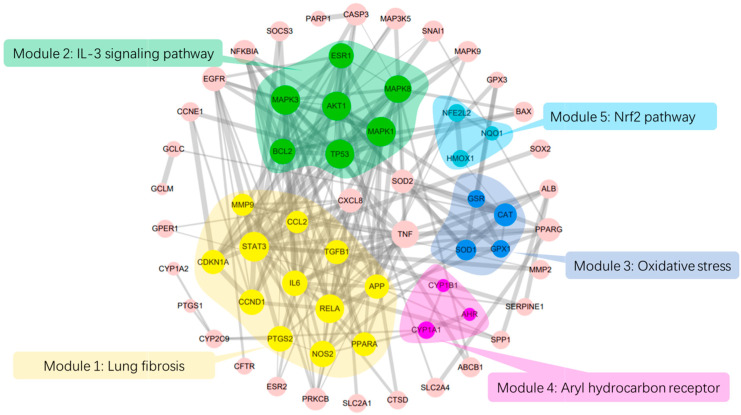
PPI network analysis and module analysis interaction targets. The size of the circles represents node degree value; the thickness of the edges represents combined score between two nodes.

A total of 1776 lung disease-related genes were collected through the DisGeNET database. A total of 135 naringenin-related targets were obtained from CTD database. Naringenin-related genes were mapped to the lung disease-related genes to obtain 69 intersection genes (Figure 1A), which were treated as target genes of naringenin for against lung diseases. The 69 target genes were used to perform PPI analysis, and the PPI network is shown in Figure 1B. The PPI data were imported into the Cytoscape software to calculate the topological parameters of the network. Our results indicated that the PPI network includes 62 nodes (seven target genes have no interaction with others) and 189 edges. The average node degree is 6.10, and the top ten degree-ranked targets are STAT3, TP53, RELA, AKT1, MAPK1, MAPK3, MAPK8, IL6, TNF, and APP, indicating their important roles for the network. The MCODE plugin was used to analyze the function module of the PPI network, and a total of five corresponding meaningful modules were identified (Figure 2). Modules 1 to 5 are most closely linked to the lung fibrosis pathway, IL-3 signaling pathway, oxidative stress pathway, Aryl hydrocarbon receptor pathway, and Nrf2 pathway, respectively.

### 2.2. GO and Pathway Enrichment Analysis

**Figure 3:**
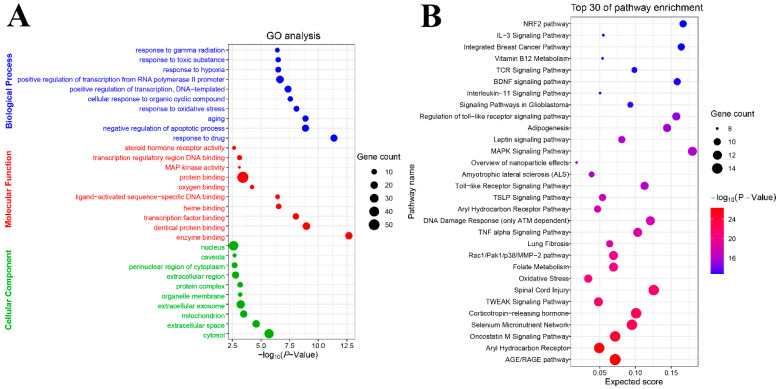
Gene Ontology (GO) and Pathway enrichment analysis of the key targets. GO terms analysis of the 69 target genes containing three aspects, including biological process, molecular function, and cellular component (**A**). Pathway enrichment analysis of the 69 target genes (**B**).

The results of GO analysis were described in terms of biological process (BP), molecular function (MF), and cellular component (CC). The results show that 144 BPs (Table S1 in the Supplemental files), 35 MFs (Table S2 in Supplemental files), and 19 CCs (Table S3 in Supplemental files) enriched for these targets were recognized as significant *p* < 0.05. An overview of the GO analysis was explored with the top 10 remarkably enriched terms in the BP, MF, and CC (Figure 3A). According to the results of pathway enrichment, 147 target-related pathways were found in the Wikipathways databases (Table S4 in Supplemental files). The top 30 Wikipathways enrichments are shown in Figure 3B. Among the top pathways related to oxidative stress include AGE/RAGE pathway, selenium micronutrient network, oxidative stress, amyotrophic lateral sclerosis, overview of nanoparticle effects, and Nrf2 pathway. The inflammation-related pathways include TWEAK signaling pathway, TNF alpha signaling pathway, MAPK signaling pathway, toll-like receptor signaling pathway, interleukin-11 signaling pathway, regulation of toll-like receptor signaling pathway, and IL-3 signaling pathway.

### 2.3. Effects of CSE and Naringenin on Cell Viability

To further strengthen the evidence for these findings, we conducted an in vitro experiment in BEAS-2B cells stimulated by CSE. We detected dynamic changes of the mRNA expressions of Nrf2, HO-1, and NQO1; the levels of total protein and nuclear protein of Nrf2; the activity of SOD; and the levels of IL-8 and TNF-α.

**Figure 4:**
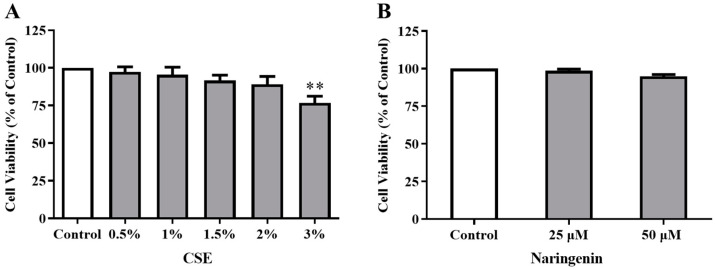
Effects of CSE (**A**) and naringenin (**B**) on cell viability of Beas-2B cells by MTT assays. Results are presented as the percentage of absorbance to the control group. Data are expressed as the means ± SD of five independent experiments for each data point (** *p* < 0.01 vs. Control).

To verify the appropriate dose of CSE used in the study, we firstly investigated the toxicity of CSE (0.5%, 1%, 1.5%, 2%, and 3%) on the cell proliferation of BEAS-2B cells using an MTT assay. As shown in Figure 4A, CSE from 0.5% to 2% did not cause any detrimental changes, while 3% CSE suppressed cell proliferation on day 6. Therefore, a 2% dose of CSE was used in the subsequent experiments. Subsequently, we tested the cytotoxicity of naringenin. Since naringenin with concentrations of 25 and 50 µM did not cause any significant changes in the viability of BEAS-2B cells (Figure 4B), we selected these two treatment concentrations throughout this study.

### 2.4. Effect of Naringenin on CSE-Induced IL-8 and TNF-α Levels

**Figure 5:**
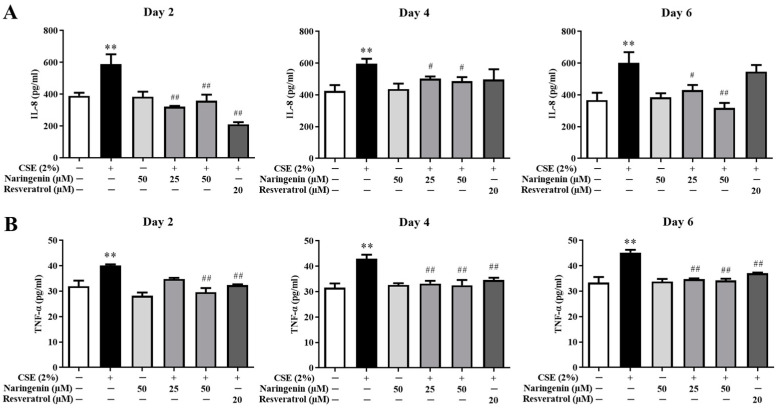
The effects of naringenin on CSE-induced IL-8 (**A**) and TNF-α (**B**) levels in BEAS-2B cells. Data are expressed as the means ± SD. ** *p* < 0.01 vs. Control group; ^#^ *p* < 0.05, ^##^ *p* < 0.01 vs. CSE alone group.

The IL-8 and TNF-α levels in the culture supernatant were measured on days 2, 4, and 6. As shown in Figure 5, the levels of IL-8 and TNF-α in the CSE alone group were significantly increased compared with the control group, demonstrating that CSE led to continuous inflammation in BEAS-2B cells. The groups co-treated different doses of naringenin (25, 50 µM) with CSE stimulation showed a significant decrease in the IL-8 and TNF-α levels on days 2, 4, and 6 compared with the CSE alone group.

### 2.5. Effect of Naringenin on CSE-Induced Nrf2 mRNA and Protein Expression

**Figure 6:**
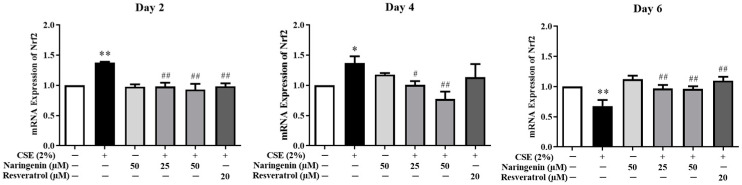
The effect of naringenin on CSE-induced mRNA expression of Nrf2 in BEAS-2B cells. The expression value was normalized by the housekeeping gene β-actin. Data are expressed as the means ± SD of at least three independent experiments for each data point. * *p* < 0.05 vs. Control group, ** *p* < 0.01 vs. Control group; ^#^ *p* < 0.05 vs. CSE alone group, ^##^ *p* < 0.01 vs. CSE alone group.

**Figure 7:**
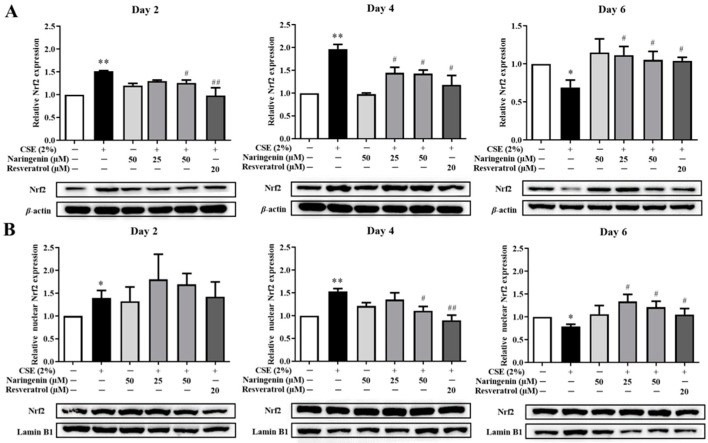
The effect of naringenin on CSE-induced total (**A**) and nuclear (**B**) protein expression of Nrf2 in BEAS-2B cells. The expression value was normalized by the housekeeping gene β-actin and lamin B1 respectively. Data are expressed as the means ± SD of at least three independent experiments for each data point. * *p* < 0.05, ** *p* < 0.01 vs. Control group; ^#^ *p* < 0.05, ^##^ *p* < 0.01 vs. CSE alone group.

Nrf2 is a critical transcription factor in the antioxidant defense system. To investigate the effect of naringenin on CSE-induced Nrf2 expression, the mRNA expression of Nrf2 was measured by RT-qPCR, and protein expression was quantified by Western blot on days 2, 4, and 6. As shown in Figure 6, the CSE-induced Nrf2 mRNA expression was significantly increased on days 2 and 4, whereas it was decreased on day 6 compared with the CSE alone group. The groups co-treated with naringenin (25, 50 µM) and CSE showed a significant decrease in Nrf2 mRNA expression on days 2 and 4, but an increase on day 6, compared with the CSE alone group. The blotting bands of both total (Figure 7A) and nuclear (Figure 7B) Nrf2 markedly increased in the CSE alone group on days 2 and 4, but decreased on day 6. Naringenin (25 and 50 µM) was able to downregulate the CSE-induced total Nrf2 protein increase on days 2 and 4, while upregulating the protein expression on day 6. The variation trend of Nrf2 nucleoprotein expression was consistent with that of its total protein expression.

### 2.6. Effects of Naringenin on CSE-Induced mRNA Expression of HO-1 and NQO1

**Figure 8:**
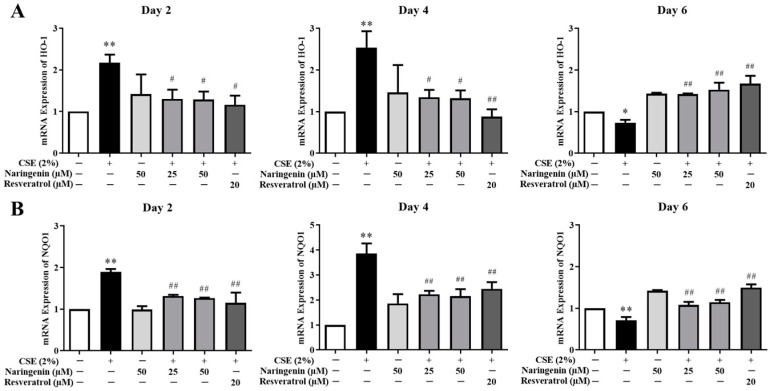
The effect of naringenin on CSE-induced mRNA expression of HO-1 (**A**) and NQO1 (**B**) in BEAS-2B cells. The expression value was normalized by the housekeeping gene *β*-actin. Data are expressed as the means ± SD of at least three independent experiments for each data point. * *p* < 0.05, ** *p* < 0.01 vs. Control; ^#^ *p* < 0.05, ^##^ *p* < 0.01 vs. CSE alone group.

To determine whether naringenin is involved in the expression of antioxidant-related genes, we measured the expressions of HO-1 and NQO1 by RT-qPCR on days 2, 4, and 6. The results showed that the mRNA expressions of HO-1 and NQO1 increased in BEAS-2B cells treated with CSE on days 2 and 4, whereas they decreased on day 6 (Figure 8). The mRNA expressions of HO-1 and NQO1 were downregulated when co-treated with naringenin (25 and 50 µM) on days 2 and 4 but upregulated on day 6.

### 2.7. Effect of Naringenin on CSE-Induced SOD Activity

**Figure 9:**
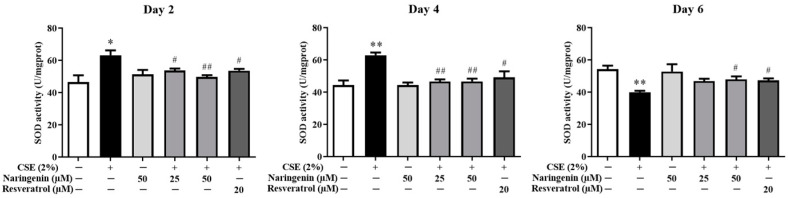
The effect of naringenin on CSE-induced SOD activity in BEAS-2B cells. Data are expressed as the means ± SD of at least three independent experiments for each data point. * *p* < 0.05, ** *p* < 0.01 vs. Control group; ^#^ *p* < 0.05, ^##^ *p* < 0.01 vs. CSE alone group.

As shown in Figure 9, the CSE-induced SOD activity was significantly upregulated on days 2 and 4, whereas is was downregulated on day 6. The groups co-treated with naringenin (25 and 50 µM) and CSE showed a remarkable decrease in SOD activity on days 2 and 4, but an increase on day 6 compared with the CSE alone group.

## 3. Discussion

The potential lung health benefits of naringenin studied here are mainly derived on the basis of bioinformatic and in vitro studies. Lung fibrosis pathway is most closely linked to events in a putative Adverse Outcome Pathway for lung fibrosis [19]. Patients with fibrotic interstitial lung disease have the potential to develop a progressive pattern characterized by self-sustaining fibrosis, weakened lung function, worsening quality of life, and early death [20]. Increasing small-airway fibrosis is a key factor of disease progression in COPD, and is presumed to originate from chronic inflammation [21]. Functional module 1 is closely linked to lung fibrosis pathway, which may contribute to the protection offered by naringenin against lung fibrosis in patients with lung disease. Functional module 2 is most closely linked to IL-3 signaling pathway. IL-3 signaling pathway is a glycoprotein involved in the process of hematopoiesis, as well as the proliferation and differentiation of pluripotent hematopoietic stem cells, progenitor cells, and their mature progeny [22]. It has been reported that hematopoietic progenitor cell levels were reduced in subjects with COPD and correlated with emphysema phenotype and severity of obstruction [23]. Naringenin may protect the maintenance of the capillary endothelium by inhibiting the reduction of HPCs, thereby contributing to alleviating the pathogenesis of COPD. Functional module 3 is linked to oxidative stress pathway. There is now substantial evidence that oxidative stress plays an important role in the pathogenesis of various lung disorders such as asthma, COPD, acute lung injury, and lung cancer [24]. A particularly remarkable etiological factor that drives COPD pathogenesis is oxidative stress, which occurs in the pulmonary milieu after long-term exposure to cigarette smoke or air pollution. Accumulating oxidative stress is an important driving mechanism in COPD, with increased ROS exposure due to cigarette smoke or environmental pollutants, as well as endogenously from activated inflammatory cells such as macrophages and neutrophils [25]. It has been shown that dietary naringenin could activate PPARα transcription factor and upregulate its fatty acid oxidation target genes [26]. The strong anti-oxidative activity of naringenin may lead to protective effects on lung health. Functional module 4 is most closely linked to Aryl hydrocarbon receptor (AHR) pathway. AHR pathway is reportedly involved in the pathogenesis of certain inflammatory lung diseases such as asthma, COPD, and silicosis [27]. AHR is a potential therapeutic target in regulating inflammation during acute and chronic respiratory diseases [28]. AHR activation may have an effect on the inflammation phase in the pathology of COPD [29]. In addition, crosstalk of AHR signaling with other ligand-activated transcription factors such as PPARs has been confirmed [30]. It has been shown that naringenin could induce regulatory T cells via an AHR-mediated pathway [31]. Thus, the regulation of AHR pathway by naringenin is possibly beneficial for protecting lung health. Functional module 5, encompassing three genes (NQO1, HMOX1, and NFE2L2), is linked to Nrf2 pathway. Factor E2-related factor (Nrf2, Gene name: NFE2L2), an important transcription factor of oxidative stress, plays a pivotal role in the regulation of multiple cytoprotective and antioxidant genes, and is considered to be a potential target for lung disease treatment. Through binding to antioxidant response elements on the target promoter regions, Nrf2 acts as a master regulator of genes encoding antioxidant enzymes, proteins and detoxifying enzymes [32,33]. Nrf2 is also implicated in lung cancer, acting as a tumor suppressor and promoter [34]. Nrf2 is significantly associated with COPD in smokers [35]. Thus, Nrf2 pathway may play an important role in the protection offered by naringenin against lung diseases. These multiple pathways interacting with each other together may be involved in the protection offered by naringenin against the damage in the lung caused by cigarette smoke.

We gained insights into the active mechanisms of naringenin for protecting lung health by using the present integrative system bioinformatic approach, but this method needs further experimental verification. In order to confirm the speculation regarding the active mechanisms experimentally, the effects of naringenin on Nrf2 pathway in BEAS-2B cells stimulated by CSE were chosen for testing. The mRNA and protein levels of Nrf2 expression decreased in the lung of patients with COPD [36,37], while cigarette smoke induced Nrf2 activation in the early stage [38,39]. Moreover, in both short-term and long-term smokers, the mRNA expression of Nrf2 in alveolar macrophages of older current smokers was lower than that of non-smokers [39], while other studies showed the CSE-induced enhancement of Nrf2 [37]. Pasini et al. reported that mild–moderate ex-smokers with COPD may be able to counteract oxidative stress by increasing the expression of Nrf2/ARE in peripheral blood mononuclear cells [40]. Recently, they found that the expression of Nrf2/ARE would decline in the oxidative stress-induced progression of COPD [41]. Thus, it is suggested that a dynamic process of oxidant/antioxidant imbalance induced by long-term CS occurs in lung disease patients. Considering that certain lung diseases, such as COPD, are chronic diseases that take decades to develop, we investigated the dynamic changes of inflammation and oxidant/antioxidant system induced by CSE and the effects of naringin on these changes in BEAS-2B cells. To date, there have been relatively few studies investigating the effects of long-term CS exposure on dynamic changes in inflammation and oxidative stress in lung. The airway epithelial cells would first come into contact with cigarette smoke after it comes into the respiratory system [42]. Usually, when studying CS-induced damage in the lung, investigating how the dynamic process of inflammation and oxidant/antioxidant system changes in BEAS-2B cells with long-term CS stimulation is useful for understanding the pathogenesis of CS-induced lung diseases.

In the early stages of CSE stimulation, the production of Nrf2 increased in response to oxidative damage; for long-term CSE stimulation, the mechanism of decline of Nrf2 remains to be elucidated. The expression of Nrf2 can reportedly be controlled epigenetically via promoter methylation [43]. Previous studies have reported that the decrease of Nrf2 in alveolar macrophages or lung tissues is negatively regulated by Bach-1, Kelch-like ECH-associated protein 1, and c-Myc [44]. It has been shown that impaired Wnt signaling contributed to Nrf2 decline by modulating GSK3b activity [45]. In this study, continuous inflammation would occur and be sustained after CSE stimulation in BEAS-2B cells. Taken together, these results indicate that there are dynamic changes of the inflammation and antioxidant system in BEAS-2B cells treated with CSE, which can model the milieu in the lung with CS exposure and be used to investigate the effects of naringenin on these changes.

Naringenin treatment was able to regulate the expression of mRNA and protein of Nrf2, the mRNA expressions of HO-1 and NQO1, and the activity of SOD to normal throughout the experiment. This suggests that naringenin can balance the oxidative/antioxidant system by regulating Nrf2 and its downstream genes to protect against the oxidative damage caused by CS stimulation, which preliminarily validates the involvement of Nrf2 pathway in the protection offered by naringenin against the damage in the lung induced by CS. To our knowledge, this is the first study reporting the effects of naringin on the dynamic changes of the oxidant/antioxidant system in BEAS-2B cells with CSE stimulation.

There is a complete set of antioxidant systems in the healthy lung, protecting against oxidative stress injury induced by CS exposure. On account of the protective effect on the respiratory system against oxidative lung and airway diseases, Nrf2 could reportedly be an attractive therapeutic target for prevention strategies and clinical therapies in respiratory illnesses [46,47]. It has also been reported that isoliquiritigenin was able to attenuate CS-Induced COPD in mice by inhibiting oxidative stress via the regulation of the Nrf2 [48]. HO-1, NQO1, and SOD are the antioxidant genes regulated by Nrf2 binding to antioxidant response elements [49]. These genes play important roles in response to oxidative stress and have cytoprotective effects [50]. Therefore, Nrf2 is considered to be a potential therapeutic target in COPD, because it can attenuate CS-induced oxidative stress by triggering a series of cytoprotective defense mechanisms [51]. Previous studies have shown that naringenin has good antioxidative activity, but the effects of naringenin on dynamic changes of oxidative stress that occurred with CS stimulation are usually overlooked. This result strengthens the evidence that naringenin can protect lung health through Nrf2 pathway.

Our results suggest that naringenin could markedly decrease the release of IL-8 and TNF-α, protecting cells from CSE-induced inflammatory reaction. IL-8 and TNF-α are essential inflammatory cytokines of pulmonary illnesses [52,53]. Clinical studies have demonstrated the correlation between pulmonary illnesses and the content of IL-8 and TNF-α [54], and strong correlations have been found between the number of sputum cells (macrophages and neutrophils) and proinflammatory cytokine levels (IL-8 and TNF-α) [55], suggesting that IL-8 and TNF-α are suitable for evaluating the inflammatory reaction in BEAS-2B cells. Although the nature of this inflammation in lungs from patients with inflammatory lung diseases has been well described, it is still uncertain how dynamic changes of inflammation relate to disease progression. The present study is helpful for understanding this better.

There is a limitation to the current study. Our investigation was based on bioinformatic analysis and an in vitro validation experiment. However, the activities of aglycone and its metabolites are not always consistent with each other [56]. Thus, the present results should therefore be treated as preliminary, and an in vivo study needs to be performed to explore whether naringenin and its metabolites exhibit similar effects.

## 4. Materials and Methods

### 4.1. Data Sources and Search Strategy

To collect potential targets of naringenin, we inputted “naringenin” into the “search-box” on Toxicogenomics Database (CTD, http://ctdbase.org/) [57]. To collect potential targets of lung diseases, “lung diseases” and “pulmonary diseases” were searched as the keywords in a database of gene-disease associations (DisGeNET, http://www.disgenet.org/).

### 4.2. Network Construction and Analysis

The naringenin-related genes were mapped to the lung disease-related genes to obtain the intersection genes of both, which can be treated as the potential targets of naringenin for treating lung diseases. Protein–protein interaction (PPI) analysis was performed on the intersection target genes by inputting official gene symbols into the “Multiple Proteins” search on the String website (https://string-db.org/) [58], with organism species set to “Homo sapiens” and a confidence score > 0.9. The PPI data were imported to Cytoscape software (version 3.7.2 Boston, MA, USA) [59] to perform network analysis using a Network Analyzer tool. The functional modules of the PPI network were identified using the molecular complex detection (MCODE) plugin.

### 4.3. Gene Ontologies and Pathway Enrichment Analysis

To identify the gene ontologies (GO) of the target genes, the David database (https://david.ncifcrf.gov) was used. To identify pathways that are significantly associated with the target genes, the GeneTrail2 web tool (https://genetrail2.bioinf.uni-sb.de/, version 1.6) was used to perform a Wikipathway enrichment analysis, and the parameters were set as described in the previous study [60].

### 4.4. Reagents

Naringenin, resveratrol, and 3-(4,5-dimethylthiazol-2-yl)-2,5-diphenyl-tetrazolium bromide (MTT) were purchased from Sigma-Aldrich (St. Louis, MO, USA). The antibiotics/antimycotics solutions (100 U/mL penicillin, 100 µg/mL streptomycin, and 0.25 µg/mL amphotericin B) were purchased from Gibco (Grand Island, NY, USA). The ELISA kits for IL-8 and TNF-α were purchased from USCNK (Wuhan, China). All primers for RT-qPCR were synthesized by Shanghai Generay Biotech Co., Ltd. (Shanghai, China). Rabbit anti-Nrf2 monoclonal antibody was purchased from Abcam (Cambridge, MA, USA). Rabbit anti-β-actin monoclonal antibody was purchased from Bioworld Technology (Bloomington, MN, USA). The secondary antibody was purchased from Promega (Madison, MI, USA). The SOD assay kit was purchased from Nanjing Jiancheng Bioengineering Institute (Nanjing, Jiangsu, China). 

### 4.5. Cigarette Smoke Extract Preparation

Cigarette smoke extract (CSE) was prepared with a modification according to the previous study [61]. Commercial cigarettes (Cocopalm, Guangzhou cigarette factory, Guangdong Province, tar concentration 12 mg) were smoked via a peristaltic pump (bt100-2j, LongerPump, Baoding, China) at a speed of 100 revolutions per minute. Four cigarettes were bubbled continuously through 10 mL of phosphate-buffered saline (PBS). The extract was filtered through a 0.22-μm pore filter (Millipore, Billerica, MA, USA) and regarded as 100% CSE.

### 4.6. Cell Viability Assay

BEAS-2B, a human bronchial epithelial cell line, was purchased from JENNIO Biological Technology Co, Ltd. (Guangzhou, China). BEAS-2B cells were maintained in DMEM (Hyclone, Logan, UT, USA) containing 10% fetal bovine serum (Lonsera, Montevideo, Uruguay) and 1% penicillin/streptomycin in a humidified atmosphere of 5% CO_2_ at 37 °C. Cell viability was evaluated by MTT assay as previously described [62]. To evaluate the effect of CSE or naringenin on cell viability, Beas-2B cells were preincubated in a 96-well and treated with various concentrations of CSE (0.5, 1, 1.5, 2, and 3%) or naringenin (25 and 50 µM) for 6 days at 37 °C. Control wells contained medium without drug. Media were changed every other day. After 6 days, 20 μL MTT dissolved in phosphate-buffered saline at 2.5 mg/mL was added to each well. After incubation for 4 h, medium was discarded and 250 μL of dimethyl sulfoxide (DMSO) was added to dissolve formazan crystals. Optical density was measured at 490 nm using the microplate reader (Biotek Epoch, Winooski, VT, USA). Cell viability was calculated as the percentage of the optical density relative to that of control sample.

### 4.7. Cell Treatment

Cells were preincubated in 6-well plates and grown to adherence, and then treated with or without 2% CSE, naringenin (25 or 50 µM), and resveratrol (20 µM) for 6 days. Control wells contained medium without the drug. Media were changed every other day. Three time points (days 2, 4, and 6) were selected to collect samples that were used for the subsequent experiments.

### 4.8. Measurement of IL-8, TNF-α, and SOD Levels

The levels of IL-8 and TNF-α in cell supernatant were detected using ELISA kits (all from Nanjing Jiancheng Bioengineering Institute, Nanjing, Jiangsu, China), according to the manufacturer’s instructions. Total protein was extracted from the cells using the Cell Lysis Buffer (10×) (CST, Boston, MA, USA). In addition, the SOD activity assay was performed using a commercial kit (Nanjing Jiancheng Bioengineering Institute, Nanjing, Jiangsu, China) according to the standard protocol.

### 4.9. RT-qPCR

The mRNA expressions of Nrf2, HO-1, and NQO1 were measured by RT-qPCR as previously described [63]. Total RNA was isolated from Beas-2B cells using RNAiso Plus (TaKaRa, Shiga, Japan). GoScript TM Reverse Transcription System (Promega, Madison, WI, USA) was used to synthesize cDNA from total RNA according to the manufacturer’s instructions. Samples were quantified by NanoDrop 2000 (Thermo, Pittsburgh, PA, USA). The RT-qPCR was performed to quantitatively determine the mRNA expressions of Nrf2, HO-1, NQO1, and β-actin using GoTaq^®^ qPCR Master Mix (Promega, Madison, WI, USA). Primer sequences are shown in Table 1. The RT-qRCR cycles were: 10 min at 95 °C, 45 cycles of 10 s at 95 °C, 20 s at 60 °C, 20 s at 72 °C, and one cycle of 5 s at 95 °C, 60 s at 65 °C, 30 s at 97 °C, finally 30 s at 40 °C. The Roche LightCycle 480 System (Roche, Mannheim, Germany) was used to monitor and analyze the fluorescence signal. For the data analysis, the comparative threshold cycle value for β-actin was used to normalize variations of gene expression in the RT-qPCR.

### 4.10. Western Blotting Analysis

Western blot analysis of nuclear and total protein of Nrf2 was performed as previously described with slight modifications [64]. Total cellular protein was prepared using the Cell Lysis Buffer (10×) (CST, USA), while nuclear protein was extracted using NE-PER™ Nuclear and Cytoplasmic Extraction Reagents (Thermo, USA). Samples were boiled for 10 min with SDS-PAGE (5×) loading buffer. After being loaded onto a 10% SDS-PAGE gel for electrophoresis, resultant samples were transferred onto PVDF membranes (Millipore, USA). The membranes were blocked with TBST containing 5% skim milk for 1 h at room temperature and then incubated with diluted primary antibodies overnight at 4 °C. After three additional washes (10 min each) with TBST, the blots were incubated with diluted HRP-conjugated secondary antibody (1:2500, Promega, Madison, USA) for 1 h at room temperature. β-actin and Lamin B1 were used as the internal control for total and nuclear protein expression, respectively. Signals were detected using Immobilon Western Chemiluminescent HRP Substrate (ECL) (Millipore, USA) and analyzed using Image J 1.42q software (National Institute of Health, Bethesda, MD, USA).

### 4.11. Statistical Analysis

The results in this study were presented as the mean ± SD from three individual measurements performed in triplicate. Statistical significance was determined by one-way ANOVA analysis. A value of *p* < 0.05 was considered statistically significant.

## 5. Conclusions

Taken together, naringenin exhibits protective effects on lung health, probably through the inhibition of inflammation and suppression of oxidative stress based on a multi-pathway network, mainly including oxidative stress pathway, Nrf2 pathway, lung fibrosis, IL-3 signaling pathway, and Aryl hydrocarbon receptor pathway. This is the first study reporting the dynamic changes of inflammation and oxidant/antioxidant system caused by CSE in BEAS-2B cells. The results indicated that the early stage of cigarette smoking can trigger an inflammatory response and activate the antioxidant system. However, with persistent smoke stimulation, inflammatory response in cells continued, and the synthesis of antioxidant products showed a progressive decline, and oxidation resistance gradually weakened, ultimately leading to an imbalance of oxidant/antioxidant system. We found that naringenin can inhibit the persistent inflammation caused by CSE and balance the oxidant/antioxidant system by regulating Nrf2 and its downstream genes, partially validating that the Nrf2 pathway is involved in the protection offered by naringenin against damage in the lung caused by CS. Our data suggest that naringenin shows potential for use in the management of lung health.

## Figures and Tables

**Table 1:** RT-qPCR primer sequence.

Gene	Direction	Primer Sequences
β-actin	Forward	CCTGTACGCCAACACAGTGC
	Reverse	ATACTCCTGCTTGCTGATCC
Nrf2	Forward	CAGCCACTTTTATTCTTCCCC
	Reverse	CAGCCACTTTTATTCTTCCCC
HO-1	Forward	CTGCAGGAACTGAGGATGCTG
	Reverse	CCAGCAACAAAGTGCAAGATTC
NQO1	Forward	CGTTTCTTCCATCCTTCCAGG
	Reverse	CGCAGACCTTGTGATATTCCAG

## Supplementary Materials

The following are available online, Table S1: Biological process analysis, Table S2: B Molecular function analysis, Table S3: Cellular component analysis, Table S4: Wikipathways enrichment results.
