# Supplementary material for: Beneficial Effects of Naringenin in Cigarette Smoke-Induced Damage to the Lung Based on Bioinformatic Prediction and In Vitro Analysis
Source: Molecules. 2020 Oct 14;25(20):4704. doi: 10.3390/molecules25204704 (PMC7587370; doi:10.3390/molecules25204704)
Supplement: Supplementary file 1 [file molecules-25-04704-s001.pdf]

Table S1. Biological process analysis.

Number	Term	Count	Adjust p-value
1	GO:0042493~response to drug	18	4.45E-12
2	GO:0043066~negative regulation of apoptotic process	18	1.22E-09
3	GO:0007568~aging	13	1.22E-09
4	GO:0006979~response to oxidative stress	11	7.59E-09
5	GO:0071407~cellular response to organic cyclic compound	9	2.66E-08
6	GO:0045893~positive regulation of transcription, DNA-templated	17	4.09E-08
7	GO:0045944~positive regulation of transcription from RNA polymerase II promoter	21	2.13E-07
8	GO:0001666~response to hypoxia	11	2.95E-07
9	GO:0009636~response to toxic substance	9	2.95E-07
10	GO:0010332~response to gamma radiation	7	3.44E-07
11	GO:0043065~positive regulation of apoptotic process	13	3.44E-07
12	GO:0031663~lipopolysaccharide-mediated signaling pathway	7	3.54E-07
13	GO:0032355~response to estradiol	9	3.54E-07
14	GO:0098869~cellular oxidant detoxification	8	1.36E-06
15	GO:0045429~positive regulation of nitric oxide biosynthetic process	7	1.90E-06
16	GO:0042542~response to hydrogen peroxide	7	5.13E-06
17	GO:0046677~response to antibiotic	6	1.53E-05
18	GO:0071356~cellular response to tumor necrosis factor	8	2.43E-05
19	GO:0006954~inflammatory response	12	2.43E-05
20	GO:0008631~intrinsic apoptotic signaling pathway in response to oxidative stress	5	2.43E-05
21	GO:0071222~cellular response to lipopolysaccharide	8	2.55E-05
22	GO:0071260~cellular response to mechanical stimulus	7	2.79E-05
23	GO:0000302~response to reactive oxygen species	6	3.15E-05
24	GO:0006805~xenobiotic metabolic process	7	4.48E-05
25	GO:0001934~positive regulation of protein phosphorylation	8	4.74E-05
26	GO:0043524~negative regulation of neuron apoptotic process	8	5.84E-05
27	GO:0010628~positive regulation of gene expression	10	5.84E-05
28	GO:0035902~response to immobilization stress	5	7.46E-05
29	GO:0006468~protein phosphorylation	12	1.03E-04
30	GO:0071456~cellular response to hypoxia	7	1.22E-04
31	GO:0051090~regulation of sequence-specific DNA binding transcription factor activity	5	1.41E-04
32	GO:0055114~oxidation-reduction process	13	1.82E-04
33	GO:0008285~negative regulation of cell proliferation	11	1.82E-04
34	GO:0045471~response to ethanol	7	1.82E-04
35	GO:0048661~positive regulation of smooth muscle cell proliferation	6	1.84E-04
36	GO:0050665~hydrogen peroxide biosynthetic process	4	2.12E-04
37	GO:0097267~omega-hydroxylase P450 pathway	4	2.12E-04
38	GO:0097421~liver regeneration	5	2.13E-04
39	GO:0051384~response to glucocorticoid	6	2.40E-04
40	GO:0008217~regulation of blood pressure	6	2.40E-04
41	GO:0032869~cellular response to insulin stimulus	6	5.26E-04

42	GO:0045454~cell redox homeostasis	6	5.26E-04
43	GO:0051092~positive regulation of NF-kappaB transcription factor activity	7	5.61E-04
44	GO:0006809~nitric oxide biosynthetic process	4	6.00E-04
45	GO:0010629~negative regulation of gene expression	7	6.34E-04
46	GO:0006974~cellular response to DNA damage stimulus	8	6.72E-04
47	GO:0035729~cellular response to hepatocyte growth factor stimulus	4	6.97E-04
48	GO:1902176~negative regulation of oxidative stress-induced intrinsic apoptotic signaling pathway	4	6.97E-04
49	GO:0007566~embryo implantation	5	7.33E-04
50	GO:0045599~negative regulation of fat cell differentiation	5	7.33E-04
51	GO:0014823~response to activity	5	7.90E-04
52	GO:0000122~negative regulation of transcription from RNA polymerase II promoter	13	8.11E-04
53	GO:0008630~intrinsic apoptotic signaling pathway in response to DNA damage	5	0.001085215
54	GO:0055093~response to hyperoxia	4	0.001137354
55	GO:0009408~response to heat	5	0.001137354
56	GO:0070141~response to UV-A	3	0.001290742
57	GO:0019373~epoxygenase P450 pathway	4	0.00130146
58	GO:0008637~apoptotic mitochondrial changes	4	0.001514436
59	GO:0051091~positive regulation of sequence-specific DNA binding transcription factor activity	6	0.001678028
60	GO:0042744~hydrogen peroxide catabolic process	4	0.001717311
61	GO:0070374~positive regulation of ERK1 and ERK2 cascade	7	0.001847235
62	GO:0043401~steroid hormone mediated signaling pathway	5	0.001961259
63	GO:0070301~cellular response to hydrogen peroxide	5	0.001961259
64	GO:0010165~response to X-ray	4	0.002162305
65	GO:0045766~positive regulation of angiogenesis	6	0.002346581
66	GO:0009611~response to wounding	5	0.002772057
67	GO:0030308~negative regulation of cell growth	6	0.002894082
68	GO:0046686~response to cadmium ion	4	0.002995498
69	GO:0043627~response to estrogen	5	0.002995498
70	GO:0018105~peptidyl-serine phosphorylation	6	0.003227561
71	GO:0035690~cellular response to drug	5	0.003672246
72	GO:0033138~positive regulation of peptidyl-serine phosphorylation	5	0.003829061
73	GO:0090200~positive regulation of release of cytochrome c from mitochondria	4	0.003857497
74	GO:0000060~protein import into nucleus, translocation	4	0.003857497
75	GO:0051881~regulation of mitochondrial membrane potential	4	0.003857497
76	GO:0042149~cellular response to glucose starvation	4	0.004125141
77	GO:0071549~cellular response to dexamethasone stimulus	4	0.004125141
78	GO:0045909~positive regulation of vasodilation	4	0.004125141
79	GO:0044344~cellular response to fibroblast growth factor stimulus	4	0.004512381
80	GO:0043200~response to amino acid	4	0.004858405
81	GO:0071392~cellular response to estradiol stimulus	4	0.004858405
82	GO:0001525~angiogenesis	7	0.005151688

83	GO:0042060~wound healing	5	0.00555814
84	GO:0043491~protein kinase B signaling	4	0.005653651
85	GO:0042908~xenobiotic transport	3	0.005890491
86	GO:0051897~positive regulation of protein kinase B signaling	5	0.00646533
87	GO:0010888~negative regulation of lipid storage	3	0.007653356
88	GO:0030522~intracellular receptor signaling pathway	4	0.008131979
89	GO:0018107~peptidyl-threonine phosphorylation	4	0.008131979
90	GO:1901215~negative regulation of neuron death	4	0.009280901
91	GO:0009410~response to xenobiotic stimulus	3	0.009280901
92	GO:0032930~positive regulation of superoxide anion generation	3	0.009280901
93	GO:0000165~MAPK cascade	7	0.010671584
94	GO:0008202~steroid metabolic process	4	0.010870496
95	GO:0048538~thymus development	4	0.010870496
96	GO:0043525~positive regulation of neuron apoptotic process	4	0.010870496
97	GO:0046902~regulation of mitochondrial membrane permeability	3	0.010974353
98	GO:0042593~glucose homeostasis	5	0.011402575
99	GO:0038095~Fc-epsilon receptor signaling pathway	6	0.011609599
100	GO:0002576~platelet degranulation	5	0.012027452
101	GO:0001649~osteoblast differentiation	5	0.012347245
102	GO:0042752~regulation of circadian rhythm	4	0.014652189
103	GO:0007254~JNK cascade	4	0.014652189
104	GO:0042738~exogenous drug catabolic process	3	0.014652189
105	GO:0036499~PERK-mediated unfolded protein response	3	0.014652189
106	GO:0019430~removal of superoxide radicals	3	0.014652189
107	GO:0030168~platelet activation	5	0.016642938
108	GO:0010745~negative regulation of macrophage derived foam cell differentiation	3	0.016642938
109	GO:0046685~response to arsenic-containing substance	3	0.016642938
110	GO:0071318~cellular response to ATP	3	0.016642938
111	GO:0016032~viral process	7	0.017704218
112	GO:0048146~positive regulation of fibroblast proliferation	4	0.018141626
113	GO:0050829~defense response to Gram-negative bacterium	4	0.018963651
114	GO:0006749~glutathione metabolic process	4	0.019804083
115	GO:0006959~humoral immune response	4	0.020662843
116	GO:1904707~positive regulation of vascular smooth muscle cell proliferation	3	0.020776081
117	GO:0032270~positive regulation of cellular protein metabolic process	3	0.020776081
118	GO:0060397~JAK-STAT cascade involved in growth hormone signaling pathway	3	0.020776081
119	GO:0007049~cell cycle	6	0.023279569
120	GO:0006950~response to stress	4	0.024077149
121	GO:0019221~cytokine-mediated signaling pathway	5	0.02420212
122	GO:0006915~apoptotic process	9	0.024526381
123	GO:0071276~cellular response to cadmium ion	3	0.025474351
124	GO:0070542~response to fatty acid	3	0.025474351
125	GO:0033189~response to vitamin A	3	0.028130152
126	GO:0033280~response to vitamin D	3	0.028130152

127	GO:0032868~response to insulin	4	0.029762802
128	GO:0007050~cell cycle arrest	5	0.029890917
129	GO:0043536~positive regulation of blood vessel endothelial cell migration	3	0.030628321
130	GO:0008284~positive regulation of cell proliferation	8	0.031387119
131	GO:1902895~positive regulation of pri-miRNA transcription from RNA polymerase II promoter	3	0.033171293
132	GO:0006801~superoxide metabolic process	3	0.033171293
133	GO:0071347~cellular response to interleukin-1	4	0.033519542
134	GO:0007584~response to nutrient	4	0.037141457
135	GO:0006367~transcription initiation from RNA polymerase II promoter	5	0.037141457
136	GO:0008283~cell proliferation	7	0.039736597
137	GO:0001836~release of cytochrome c from mitochondria	3	0.042142439
138	GO:0014066~regulation of phosphatidylinositol 3-kinase signaling	4	0.042142439
139	GO:0045892~negative regulation of transcription, DNA-templated	8	0.042806506
140	GO:0001503~ossification	4	0.044605343
141	GO:0045742~positive regulation of epidermal growth factor receptor signaling pathway	3	0.044641597
142	GO:0032496~response to lipopolysaccharide	5	0.046288404
143	GO:0007569~cell aging	3	0.047720232
144	GO:0006919~activation of cysteine-type endopeptidase activity involved in apoptotic process	4	0.048081092

Table S2. Molecular function analysis.

Number	Term	Count	Adjust P-value
1	GO:0019899~enzyme binding	19	1.79E-13
2	GO:0042802~identical protein binding	21	1.05E-09
3	GO:0008134~transcription factor binding	14	8.43E-09
4	GO:0020037~heme binding	10	2.70E-07
5	GO:0004879~RNA polymerase II transcription factor activity, ligand-activated sequence-specific DNA binding	7	3.38E-07
6	GO:0019825~oxygen binding	6	5.73E-05
7	GO:0005515~protein binding	54	3.65E-04
8	GO:0004707~MAP kinase activity	4	7.25E-04
9	GO:0044212~transcription regulatory region DNA binding	8	7.25E-04
10	GO:0003707~steroid hormone receptor activity	5	0.002115
11	GO:0019903~protein phosphatase binding	5	0.003053
12	GO:0042803~protein homodimerization activity	12	0.003173
13	GO:0030284~estrogen receptor activity	3	0.003173
14	GO:0004784~superoxide dismutase activity	3	0.003173
15	GO:0008144~drug binding	5	0.004636
16	GO:0016301~kinase activity	7	0.007259
17	GO:0030235~nitric-oxide synthase regulator activity	3	0.007259
18	GO:0046982~protein heterodimerization activity	9	0.00843

19	GO:0001046~core promoter sequence-specific DNA binding	4	0.010131
20	GO:0002020~protease binding	5	0.010261
21	GO:0019901~protein kinase binding	8	0.010343
22	GO:0043565~sequence-specific DNA binding	9	0.013909
23	GO:0032403~protein complex binding	6	0.01754
24	GO:0016712~oxidoreductase activity, acting on paired donors, with incorporation or reduction of molecular oxygen, reduced flavin or flavoprotein as one donor, and incorporation of one atom of oxygen	3	0.01844
25	GO:0004497~monooxygenase activity	4	0.01844
26	GO:0004672~protein kinase activity	7	0.034509
27	GO:0004601~peroxidase activity	3	0.036359
28	GO:0051087~chaperone binding	4	0.042623
29	GO:0003682~chromatin binding	7	0.044619
30	GO:0003700~transcription factor activity, sequence-specific DNA binding	11	0.044619
31	GO:0051721~protein phosphatase 2A binding	3	0.044619
32	GO:0070330~aromatase activity	3	0.044619
33	GO:0005496~steroid binding	3	0.044619
34	GO:0031625~ubiquitin protein ligase binding	6	0.049209
35	GO:0003677~DNA binding	15	0.049369

Table S3. Cellular component analysis.

Number	Term	Count	Adjust P-value
1	GO:0005829~cytosol	34	1.88E-06
2	GO:0005615~extracellular space	20	2.45E-05
3	GO:0005739~mitochondrion	18	3.10E-04
4	GO:0070062~extracellular exosome	26	5.80E-04
5	GO:0031090~organelle membrane	6	6.10E-04
6	GO:0043234~protein complex	10	6.10E-04
7	GO:0005576~extracellular region	18	0.001568467
8	GO:0048471~perinuclear region of cytoplasm	11	0.001921286
9	GO:0005901~caveola	5	0.001921286
10	GO:0005634~nucleus	36	0.002326097
11	GO:0005654~nucleoplasm	22	0.013728987
12	GO:0045121~membrane raft	6	0.013858846
13	GO:0043231~intracellular membrane-bounded organelle	9	0.013858846
14	GO:0031093~platelet alpha granule lumen	4	0.013858846
15	GO:0005789~endoplasmic reticulum membrane	11	0.014606385
16	GO:0000307~cyclin-dependent protein kinase holoenzyme complex	3	0.014919621
17	GO:0072562~blood microparticle	5	0.025187513
18	GO:0016323~basolateral plasma membrane	5	0.041518785
19	GO:0005794~Golgi apparatus	10	0.041518785

Table S4. Wikipathways enrichment results.

Number	Term	Count	Adjusted p-value
1	AGE/RAGE pathway	15	3.43E-25
2	Aryl Hydrocarbon Receptor	14	8.20E-24
3	Oncostatin M Signaling Pathway	14	3.69E-23
4	Selenium Micronutrient Network	14	1.11E-22
5	Corticotropin-releasing hormone	14	1.49E-02
6	TWEAK Signaling Pathway	12	5.99E-22
7	Spinal Cord Injury	14	1.07E-21
8	Oxidative Stress	11	2.27E-21
9	Folate Metabolism	12	1.09E-20
10	Rac1/Pak1/p38/MMP-2 pathway	12	3.48E-19
11	Lung fibrosis	10	3.59E-19
12	TNF alpha Signaling Pathway	12	3.63E-19
13	DNA Damage Response (only ATM dependent)	12	1.68E-18
14	Aryl Hydrocarbon Receptor Pathway	10	1.68E-17
15	TSLP Signaling Pathway	10	5.96E-17
16	Toll-like Receptor Signaling Pathway	11	6.26E-17
17	Amyotrophic lateral sclerosis (ALS)	9	6.48E-17
18	Overview of nanoparticle effects	8	6.48E-17
19	MAPK Signaling Pathway	12	7.36E-16
20	Leptin signaling pathway	10	3.13E-15
21	Adipogenesis	11	1.86E-14
22	Regulation of toll-like receptor signaling pathway	11	2.93E-14
23	Signaling Pathways in Glioblastoma	9	6.07E-14
24	Interleukin-11 Signaling Pathway	8	6.07E-14
25	BDNF signaling pathway	10	9.48E-14
26	TCR Signaling Pathway	9	9.64E-14
27	Vitamin B12 Metabolism	8	1.02E-13
28	Integrated Breast Cancer Pathway	10	1.41E-13
29	IL-3 Signaling Pathway	8	3.43E-13
30	NRF2 pathway	10	3.79E-12
31	Insulin Signaling	10	4.11E-12
32	Apoptosis-related network due to altered Notch3 in ovarian cancer	8	4.11E-12
33	IL-1 signaling pathway	8	4.69E-12
34	Kit receptor signaling pathway	8	5.97E-12
35	Estrogen Receptor Pathway	6	7.70E-12
36	TGF-beta Signaling Pathway	9	1.21E-11
37	Transcriptional activation by NRF2	6	1.88E-11
38	Apoptosis	8	2.64E-11
39	Apoptosis Modulation and Signaling	8	2.90E-11
40	Integrated Cancer Pathway	7	5.31E-11
41	Wnt Signaling Pathway Netpath	7	7.82E-11
42	MicroRNAs in cardiomyocyte hypertrophy	8	2.95E-10

43	Estrogen signaling pathway	6	3.38E-10
44	IL-4 Signaling Pathway	7	3.38E-10
45	RANKL/RANK Signaling Pathway	7	3.43E-10
46	Senescence and Autophagy in Cancer	8	4.59E-10
47	TGF-beta Receptor Signaling	7	5.37E-10
48	Circadian rythm related genes	9	7.75E-10
49	Bladder Cancer	6	1.19E-9
50	Nuclear Receptors in Lipid Metabolism and Toxicity	6	1.35E-9
51	IL-6 signaling pathway	6	1.37E-9
52	Osteopontin Signaling	5	1.45E-9
53	Androgen receptor signaling pathway	7	1.59E-9
54	IL-2 Signaling Pathway	6	2.15E-9
55	B Cell Receptor Signaling Pathway	7	2.30E-9
56	Structural Pathway of Interleukin 1 (IL-1)	6	3.42E-9
57	Copper homeostasis	6	5.21E-9
58	Focal Adhesion	8	5.81E-9
59	ErbB Signaling Pathway	6	1.09E-8
60	Cardiac Hypertrophic Response	6	1.09E-8
61	EBV LMP1 signaling	5	1.34E-8
62	Glutathione metabolism	5	2.42E-8
63	DNA Damage Response	6	2.45E-8
64	TCA Cycle Nutrient Utilization and Invasiveness of Ovarian Cancer	4	3.46E-8
65	IL-7 Signaling Pathway	5	3.46E-8
66	IL17 signaling pathway	5	3.84E-8
67	EGF/EGFR Signaling Pathway	7	6.61E-8
68	miRNA Regulation of DNA Damage Response	6	1.01E-7
69	PDGF Pathway	5	1.16E-7
70	Nuclear Receptors	5	2.43E-7
71	FAS pathway and Stress induction of HSP regulation	5	2.66E-7
72	IL-5 Signaling Pathway	5	2.82E-7
73	Alzheimers Disease	6	2.82E-7
74	Estrogen metabolism	4	3.33E-7
75	TP53 Network	4	4.62E-7
76	Type II diabetes mellitus	4	5.34E-7
77	TSH signaling pathway	5	7.51E-7
78	Tamoxifen metabolism	4	8.10E-7
79	Physiological and Pathological Hypertrophy of the Heart	4	8.27E-7
80	SIDS Susceptibility Pathways	6	1.03E-6
81	EPO Receptor Signaling	4	2.09E-6
82	Allograft Rejection	5	2.30E-6
83	Nanoparticle-mediated activation of receptor signaling	4	4.09E-6
84	Signaling of Hepatocyte Growth Factor Receptor	4	4.83E-6
85	Metapathway biotransformation	6	1.43E-5

86	Wnt Signaling Pathway and Pluripotency	5	1.46E-5
87	ATM Signaling Pathway	4	1.54E-5
88	Selenium Metabolism and Selenoproteins	4	2.90E-5
89	miRNAs involved in DNA damage response	4	3.34E-5
90	Tryptophan metabolism	4	3.34E-5
91	TFs Regulate miRNAs related to cardiac hypertrophy	3	4.35E-5
92	Notch Signaling Pathway	4	4.91E-5
93	Endochondral Ossification	4	6.23E-5
94	Quercetin and Nf-kB/ AP-1 Induced Cell Apoptosis	3	7.75E-5
95	G1 to S cell cycle control	4	8.45E-5
96	Oxidation by Cytochrome P450	4	8.45E-5
97	AMPK Signaling	4	9.14E-5
98	IL-9 Signaling Pathway	3	1.01E-4
99	Angiogenesis	3	1.01E-4
100	Retinoblastoma (RB) in Cancer	4	1.10E-4
101	FSH signaling pathway	3	3.08E-4
102	Signal Transduction of S1P Receptor	3	4.41E-4
103	Cytokines and Inflammatory Response	3	7.57E-4
104	Melatonin metabolism and effects	3	8.89E-4
105	Cell Cycle	4	8.89E-4
106	Extracellular vesicle-mediated signaling in recipient cells	3	8.89E-4
107	Matrix Metalloproteinases	3	1.01E-3
108	Alpha 6 Beta 4 signaling pathway	3	1.08E-3
109	Dopaminergic Neurogenesis	3	1.08E-3
110	Sulindac Metabolic Pathway	2	1.50E-3
111	Wnt Signaling Pathway	3	1.50E-3
112	Benzo(a)pyrene metabolism	2	1.73E-3
113	NLR Proteins	2	1.86E-3
114	Endoderm Differentiation	3	2.24E-3
115	Mesodermal Commitment Pathway	3	2.49E-3
116	Primary Focal Segmental Glomerulosclerosis FSGS	3	2.49E-3
117	Cori Cycle	2	2.65E-3
118	Irinotecan Pathway	2	2.75E-3
119	Dopamine metabolism	2	3.05E-3
120	Fatty Acid Omega Oxidation	2	3.36E-3
121	Osteoclast Signaling	2	5.24E-3
122	Drug Induction of Bile Acid Pathway	2	5.57E-3
123	Serotonin Receptor 4/6/7 and NR3C Signaling	2	5.57E-3
124	Serotonin Receptor 2 and ELK-SRF/GATA4 signaling	2	6.42E-3
125	PPAR Alpha Pathway	2	6.78E-3
126	Eicosanoid Synthesis	2	6.78E-3
127	IL1 and megakaryotyces in obesity	2	7.70E-3
128	MAPK Cascade	2	7.95E-3
129	T-Cell Receptor and Co-stimulatory Signaling	2	7.95E-3

130	Gastric Cancer Network 2	2	7.95E-3
131	Regulation of Actin Cytoskeleton	3	7.95E-3
132	Monoamine Transport	2	1.06E-2
133	Hypothetical Network for Drug Addiction	2	1.11E-2
134	Initiation of transcription and translation elongation at the HIV-1 LTR	2	1.28E-2
135	Prostaglandin Synthesis and Regulation	2	1.59E-2
136	Trans-sulfuration and one carbon metabolism	2	2.18E-2
137	Constitutive Androstane Receptor Pathway	2	2.40E-2
138	Type II interferon signaling (IFNG)	2	3.09E-2
139	Glycolysis and Gluconeogenesis	2	3.45E-2
140	NOD pathway	2	3.70E-2
141	Differentiation Pathway	2	3.70E-2
142	Cardiac Progenitor Differentiation	2	4.19E-2
143	Interferon type I signaling pathways	2	4.19E-2
144	Parkinsons Disease Pathway	2	2.63E-002
145	SREBP signalling	2	3.39E-002
146	Butyrate-induced histone acetylation	1	4.37E-002
147	Lidocaine metabolism	1	4.37E-002
